# Identification and Gene Fine Mapping of the Bisexual Sterility Mutant *Meiosis Abnormal Bisexual Sterility 1* in Rice

**DOI:** 10.3390/cimb46110773

**Published:** 2024-11-14

**Authors:** Yingchun Wan, Xiaoqing Liu, Nan Wang, Zhengming Zeng, Yudong Jiang

**Affiliations:** 1 Deyang Branch of Sichuan Academy of Agricultural Sciences, Luzhou Branch of National Rice Improvement Center, Southwest Key Laboratory of Rice Biology, Rice and Sorghum, Research Institute of Sichuan Academy of Agricultural Sciences, Genetics and Breeding of Ministry of Agriculture, Deyang 618000, China; wanyc1221@126.com; 2 Transgenic Plants and Safety Control Chongqing Municipal Key Laboratory, Rice Research Institute of Southwest University, Engineering Research Center of Southern Mountain Agriculture Ministry of Education, Chongqing 400716, China; lxq200010100321@163.com (X.L.);

**Keywords:** rice (*Oryza sativa* L.), bisexual sterility, meiosis, gene mapping, *MABS1*

## Abstract

Exploring the genes regulating rice fertility is of great value for studying the molecular mechanisms of rice reproductive development and production practices. In this study, we identified a sterile mutant from the mutant library induced by ethyl methanesulfonate (EMS), designated as *meiosis abnormal bisexual sterility 1* (*mabs1*). The *mabs1* mutant exhibits no phenotypic differences from the wild-type during the vegetative growth phase but shows complete sterility during the reproductive growth phase. Phenotypic observations revealed that both pollen and embryo sac fertility are lost in *mabs1*. Notably, in *mabs1*, the development of the anther inner and outer walls, tapetum degeneration, and callose synthesis and degradation all proceed normally, yet meiosis fails to form normal tetrads. Genetic analysis indicated that this mutant trait is controlled by a single recessive nuclear gene. By constructing a genetic segregation population, we successfully mapped the *MABS1* gene to a 49 kb region between primer markers Y7 and Y9 on chromosome 1. Resequencing revealed a single-nucleotide substitution in the exon of the *LOC_Os01g66170* gene, which resulted in a change from Valine to Isoleucine. Subsequent sequencing of this locus in both wild-type and *mabs1* mutants confirmed this mutation. Therefore, we have identified the gene at *LOC_Os01g66170* as a candidate for *MABS1*, a previously unreported novel gene involved in rice meiosis. Through RT-qPCR, we found that the expression levels of multiple meiosis-related genes were significantly changed in the *mabs1* mutant. Therefore, we believe that *MABS1* is also involved in the process of rice meiosis. This study lays the groundwork for a functional study of *MABS1*.

## 1. Introduction

Rice is a globally significant food crop and serves as a model plant for monocotyledonous biology, thus holding immense value for both practical applications and scientific research. The development of rice fertility is directly related to rice yield, so the exploration and analysis of genes related to rice fertility development will help increase rice production [1]. Meiosis is one of the sources of genetic diversity. By manipulating the meiosis process, crop varieties with specific traits can be cultivated. For example, by knocking out recombination inhibitors or precisely regulating proteins related to the recombination pathway, the frequency of genetic recombination can be increased, thereby enhancing the genetic diversity of crops [2]. 

The smallest unit of a rice flower is the spikelet, which contains the anther and the ovary. The anther is an important organ for the formation of male gametes, and the ovary is an organ for the formation of female gametes. Rice anthers are composed of four layers: the epidermis, the endothecium, the middle layer, and the tapetum. Microspores develop into mature pollen within the anthers, and this development is closely related to the differentiation and formation of the anther wall layers [3]. Early studies categorized the development of rice anthers into eight stages based on the morphological analysis of semi-thin sections: the formation of microspore mother cells, the meiotic division of microspore mother cells, early microspore, mid microspore, late microspore, early binucleate pollen, late binucleate pollen, and mature pollen [4]. Subsequent research further refined anther development into 14 stages, analyzing specific developmental events for each [1,5]. The fertility development of rice pollen involves a complex regulatory network with extensive gene expression control [6]. Cloned male sterility genes have been implicated in biological processes such as pollen meiosis, tapetum degradation, pollen wall formation, anther dehiscence, and pollen tube elongation [7,8].

Meiosis is a critical step in the development of sexual reproductive cells, where microspore and megaspore mother cells undergo meiosis to produce haploid microspores and megaspores, respectively, which then develop into mature pollen and embryo sacs through mitosis [9]. Over 90 meiosis-related genes have been identified in plants, with more than 50 discovered in rice, participating in key steps of meiosis such as the induction of double-strand breaks (DSBs), the resection of DSB ends, the formation of recombination intermediates, DNA synthesis, and crossover formation [10,11]. Early studies in yeast indicated that topoisomerase-like protein SPO11 induces DSBs, initiating meiotic recombination [12]. In Arabidopsis, two SPO11 homologs, AtSPO11-1 and AtSPO11-2, interact and function together, with mutations in either gene leading to abnormalities in homologous recombination during meiosis [6,13,14]. In rice, four SPO11 homologs have been identified; no phenotypes have been reported for the *OsSPO11*-3 mutant, whereas knockout mutants of *OsSPO11-1* and *OsSPO11-2* exhibit sterile phenotypes, suggesting that *OsSPO11-4* is not essential for meiotic recombination in rice [15]. In addition, more than 70 proteins have been found to be involved in various stages of meiosis in rice. *GSL5* encodes a callose synthase, which has the function of inducing meiosis in the early stage of anther development [16]. OsRAD51D and OsDMC1B are involved in DNA repair and recombination [17,18]. OsMTOPVIB participates in the assembly of meiotic spindles by changing the adhesion of sister chromatid centromeres [19]. OsPAIR1 affects chromosome homologous pairing during meiosis; the *ospair1* mutant fails to pair homologous chromosomes, with all chromosomes clustering around the nucleolus, rendering both male and female gametes infertile [20]. When *OsOSD1*, *PAIR1*, and *OsREC8*, three meiosis control genes, are simultaneously knocked out, the mutant undergoes mitosis instead of meiosis without chromosome exchange, resulting in the production of MiMe material with diploid male and female gametes identical to the parent somatic cells [21]. The *oscrc1* mutant shows normal vegetative growth but is completely sterile; OsCRC1 forms a complex with OsZEP1 and OsPAIR1 during meiosis, promoting DSB formation [22]. Rice OsCOM1 is involved in the resection of DSB ends during meiosis; the *oscom1* mutant experiences severe impairment in synaptonemal complex formation, homologous pairing, and recombination, preventing the loading of OsZEP1 and OsMER3 onto chromosomes [23]. According to the analysis of the discovered meiotic regulatory gene regulatory network, there are still meiotic genes that have not yet been discovered.

Our research team identified a rice sterility mutant, ***meiosis abnormal bisexual sterility 1*** (*mabs1*). Phenotypic characterization of the mutant revealed abnormal meiotic division and complete sterility of the pollen. The candidate gene was mapped to *LOC_Os01g66170*, with no prior reports on this gene. Therefore, the MABS1 protein identified in this study is a novel regulator of fertility in rice, laying the foundation for a deeper understanding of the regulatory network of rice reproductive development.

## 2. Materials and Methods

### 2.1. Plant Growth and Observation on Fertility

The rice (*O. sativa* L. ssp. *indica*) *mabs1* mutant was identified from a “Xida 1B” (wild-type) population mutagenized with EMS. The polymorphic variety Jinhui 10 was crossed with *MABS1* fertile plants, and the segregation population F_2_ was obtained by screening. At maturity, entire plants of the wild-type and *mabs1* mutant rice, grown simultaneously, were photographed using a Canon MARK5DIII digital camera (Canon, Tokyo, Japan) for documentation. The plant height at the highest leaf, the width at the widest leaf, and the number of effective tillers were measured, each data measurement was repeated three times to take the average, and Student’s *t*-test was performed. During the flowering stage of rice, randomly selected spikelets from the wild-type and the *mabs1* mutant at stage 13 were observed and photographed using a Nikon SMZ1500 (Nikon, Tokyo, Japan) stereomicroscope for their overall morphology. Subsequently, the lemma and palea of the spikelets were separated using tweezers, and the internal anthers, stigmas, and ovaries were observed and photographed. The anthers were placed on a slide with 1% I_2_-KI solution, crushed with tweezers to ensure full contact with the staining solution, and stained in the dark at room temperature for 1 minute. Pollen staining was then observed and photographed using an Eclipse E600 microscope (Nikon). 

### 2.2. Scanning Electron Microscopy

During the flowering stage of rice, we selected three wild-type and *mabs1* mutant plants with normal growth each, and spikelets from the wild-type and *mabs1* mutant at stages 13–14 were randomly selected. After peeling back the lemma and palea using tweezers, multiple anthers were carefully placed on a conductive adhesive-coated observation platform. The selected anthers from both the wild-type and *mabs1* mutant were opened along the anther loculus to expose the anther wall and the internal pollen grains. The overall morphology, anther wall, anther cavity, and pollen grains of the wild-type and *mabs1* mutant were observed and photographed using a Hitachi SU3500 scanning electron microscope (Hitachi, Tokyo, Japan).

### 2.3. Semi-Thin Section Observation

Semi-thin sections were prepared from spikelets of the wild-type and *mabs1* mutant at different developmental stages for observation of the anther structure [24]. The specific steps were as follows: spikelets from the wild-type and *mabs1* at different developmental stages were immersed in glutaraldehyde fixative for more than 48 h; after replacing the fixative with 0.1 M PBS, the anthers were immersed in 1% osmium tetroxide for secondary fixation; the samples were then dehydrated using a gradient series of ethanol (30%, 50%, 70%, 80%, 90%, 100%, 100%, and 100%), followed by anhydrous acetone dehydration; finally, the SPI-PON 812 resin (Shanghai Physion Instruments, Shanghai, China) embedding agent was used to gradually replace the anhydrous acetone, and the anthers were embedded in fully cured resin. After hardening, semi-thin sections were made using a Leica RM-2265 microtome (Leica, Wetzal, Germany), stained with 1% toluidine blue, and observed using an Eclipse E600 microscope (Nikon).

### 2.4. Paraffin Sectioning

Paraffin sectioning and embedding of spikelets from the wild-type and *mabs1* mutant at different developmental stages were performed [25]. The specific steps were as follows: spikelets from the wild-type and *mabs1* at different developmental stages were immersed in FAA fixative and placed in the dark at 4 °C for more than 48 h; the samples were dehydrated using a gradient series of ethanol (30%, 50%, 75%, 90%, 100%, 100%, and 100%); the dehydrated materials were cleared at room temperature using a mixture of xylene and anhydrous ethanol in a ratio of 1:3, followed by 1:1, then 3:1, and, finally, pure xylene three times; the materials were then infiltrated with a 1:1 mixture of xylene and molten paraffin, placed in a 65 °C oven for 12 h, after which the mixture was poured out and replaced with molten pure paraffin twice; then, the paraffin-embedded materials were sectioned according to the position of the materials using a Leica RM-2245 microtome (Leica). The sections were placed on slides, put in a 40 °C oven for 2 days, and then ready for subsequent staining or stored at 4 °C.

### 2.5. Callose Staining Observation

Paraffin sections from the wild-type and *mabs1* mutant at different stages were preheated on a 42 °C slide-warming platform, then placed in a staining jar, dewaxed with pure xylene, and washed three times with anhydrous ethanol. The dewaxed sections were rehydrated using a gradient series of ethanol (90%, 80%, 70%, 50%, and 30%) for 5 min each, followed by two washes with 0.1 M K-PBS for 3 min each. The sections were then stained with 0.1% aniline blue in a staining jar for 2 h in the dark at room temperature. After staining, the slides were washed twice with 0.1 M K-PBS for 5 min each in the dark at room temperature. The stained slides were mounted with 50% glycerol and observed and photographed under a fluorescence microscope using ultraviolet light.

### 2.6. Meiotic Behavior Observation

Spikelets from the wild-type and *mabs1* mutant during meiosis were fixed using Carnoy’s fixative. The fixed spikelets were placed on slides, and the anthers were dissected and chopped with a blade. A drop of acetic carmine was added to the anthers, covered with a cover slip, and quickly heated over an alcohol lamp until near boiling; then, heating was stopped. The slides were washed with 40% acetic acid to remove the acetic carmine staining solution. The slides were then frozen in liquid nitrogen, and, after removal, the cover slips were quickly removed with a blade. The anthers were dehydrated using a gradient series of ethanol (50%, 70%, 90%, and 100%). A drop of mounting medium containing DAPI was added to the anthers, covered with a cover slip, and observed and photographed under a fluorescence microscope.

### 2.7. Genetic Analysis and Fine Mapping

The F_1_ generation was obtained by crossing *MABS1* with Jinhui 10, and the F_2_ generation was obtained by the self-pollination of single plants from F_1_. The segregation ratio of the sterile phenotype in the F_2_ population was subjected to a χ^2^ test (χ^2^_3:1_, *p* > 0.05 indicates no significant difference). Preliminary and fine mapping were performed using the sterile group from the F_2_ generation. DNA was extracted from leaf samples of the mapping population using the CTAB method [3]. Polymorphic SSR (Simple Sequence Repeat) markers were developed using public rice databases Gramene (http://www.gramene.org/, accessed on 8 June 2024) and the Rice Genome Research Program (http://rgp.dna.afrc.go.jp/E/publicdata/caps/index.html/, accessed on 8 June 2024); in response to the polymorphic differences between the Xida 1B and Jinhui 10 sequences, SSR primers were designed to be evenly distributed across the 12 chromosomes of rice (Table S2). These primers were used for the molecular identification of the parental lines and a mixed gene pool of 30 F_2_ generation mutants to determine the linked loci. Further SSR primers were designed in the vicinity of the linked loci to perform molecular identification on individual plants of the parental lines and 142 F_2_ generation mutants, narrowing down the localization interval until no sequence differences were found within the interval. A mixed gene pool of 30 F_2_ generation mutants was sent to Beijing Novogene Technology Co., Ltd. (Novogene Technology, Beijing, China), where a whole-genome resequencing analysis was conducted using the second-generation Illumina PE150 platform. The IGV (version: IGV-2.8.13) software was utilized to compare sequence differences between the parental lines and *mabs1* mutants in the open reading frames within the localization interval, and PCR identification was performed in 60 F_2_ generation mutants and 60 F_2_ generation wild-type. The PCR reaction system was 12.6 μL, containing 1.25 μL 10 × PCR buffer, 1 μL 50 ng μL^−1^ DNA template, 0.75 μL 25 mmol L^−1^ MgCl_2_, 0.5 μL 2.5 mmol L^−1^ dNTPs, 8.0 μL ddH_2_O, 1.0 μL 10μmol L^−1^ primer, and 0.1 μL 5 U μL^−1^ Taq DNA polymerase. The PCR program was 94 °C for 5 min of initial denaturation; 94 °C for 30 s of denaturation, 55 °C for 30 s of annealing, 72 °C for 30 s of extension, for 35 cycles; and 72 °C for 10 min of final extension. PCR products were observed after 10% non-denaturing polyacrylamide gel electrophoresis and quick silver staining [26,27].

### 2.8. Bioinformatic Analysis

Gene sequences were retrieved using Gramene (https://www.gramene.org/, accessed on 8 August 2024) and NCBI (https://blast.ncbi.nlm.nih.gov/, accessed on 8 August 2024). Amino acid sequence alignment was performed using SnapGene (version: snapgene-6.0.2) software (https://www.snapgene.com/, accessed on 8 August 2024). A phylogenetic tree was constructed using the neighbor-joining method with MEGA X (version: MEGAX-10.1). Protein information was collected using Uniprot (https://www.uniprot.org/, accessed on 15 October 2024). The tertiary structure of MABS1 and mabs1 proteins was analyzed using AlphaFold (https://alphafold.com/, accessed on 15 October 2024) [28,29,30].

### 2.9. Subcellular Localization

The full-length coding frame of MABS1 was amplified and inserted into the transient expression vector pAN580 (35S × 2-GFPNOS) to generate the fusion protein GFP-MABS1. The fusion expression vectors were transformed into rice protoplasts in accordance with a method reported previously [25]. The empty GFP vector was used as the negative control, and the reported cell membrane localization protein TGW2 was used to link mCherry as a marker [31]. After transformation, the protoplasts were cultured overnight at 28 C in the dark, and then, the fluorescence signal was observed with an LSM800 confocal laser scanning microscope (Zeiss, Oberkochen, Germany). The primer sequences used are listed in Supplementary Table S2.

### 2.10. RT-qPCR

We extracted total RNA from the dissected roots, culms, leaves, sheaths, and spikelets of the wild-type and from the anthers of wild-type and *mabs1* mutant from stage 5 to stage 11 using an RNA extraction kit (Promega, Beijing, China). cDNA was synthesized using a reverse transcription kit (US Everbright Inc, Jiangsu, China). Quantitative primers were designed based on data from the China National Rice Research Center database (https://ricedata.cn/, accessed on 8 June 2024). We designed primers based on the specific sequences of the CDS of the gene to be analyzed, making sure that the amplified fragment size was 200–400 bp (Table S2). A real-time quantitative PCR (RT-qPCR) analysis was performed using an ABI 7500 Sequence Detection System (Thermo Fisher, Waltham, MA, USA) with an SYBR Premix Ex Taq GC Kit (Takara, Dalian, China). *OsACTIN1* was used as an endogenous control. Three replicates for each sample were analyzed. Normalized relative expression levels were calculated using the ΔΔC_t_ method [32].

## 3. Results

### 3.1. mabs1 Fertility Identification

**Figure 1:**
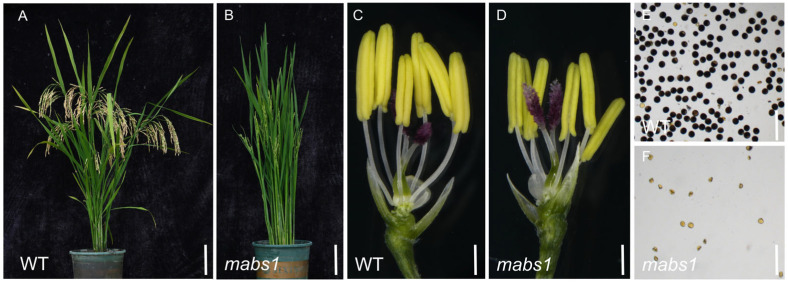
Fertility observation of wild-type Xida 1B and *mabs1*: (**A**,**B**) plants of wild-type and *mabs1*; (**C**,**D**) spikelet without lemma and palea of wild-type and *mabs1*; and (**E**,**F**) pollen I_2_-KI staining of wild-type and *mabs1*. Scale bars: 10 cm (**A**,**B**), 1 mm (**C**,**D**), and 0.1 mm (**E**,**F**).

Field observations revealed that there was no significant difference in plant height and leaf width between the *mabs1* mutant and the wild-type Xida 1B, but the effective tiller number of *mabs1* was significantly reduced and showed no seed (Figure 1A,B and Figure S1). The microscopic examination of the anthers revealed that, while wild-type anthers were plump and yellow, by contrast, *mabs1* anthers were smaller, shriveled, and lightly colored (Figure 1C,D). Staining with 1% I_2_-KI solution indicated that wild-type pollen grains were round and fully starch-filled, appearing dark brown, whereas *mabs1* pollen grains were misshapen, wrinkled, and lacked starch filling (Figure 1E,F), suggesting abnormal pollen development in *mabs1*. Staining of the pollen exine with Auramine and the intine with Calcofluor White revealed intact pollen wall structures in both wild-type and *mabs1*, indicating that wall development was not the cause of sterility (Figure S2). Since the *mabs1* mutant was completely sterile from self-pollination and had fully aborted pollen, we crossed it with wild-type pollen to assess the viability of the female gametophyte (Table S1). The lack of seed in the hybrid strain indicated that the female gametophyte of *mabs1* was aborted, suggested that *mabs1* was a male–female sterility mutant.

### 3.2. Scanning Electron Microscopy of mabs1 Mutant Anthers

**Figure 2:**
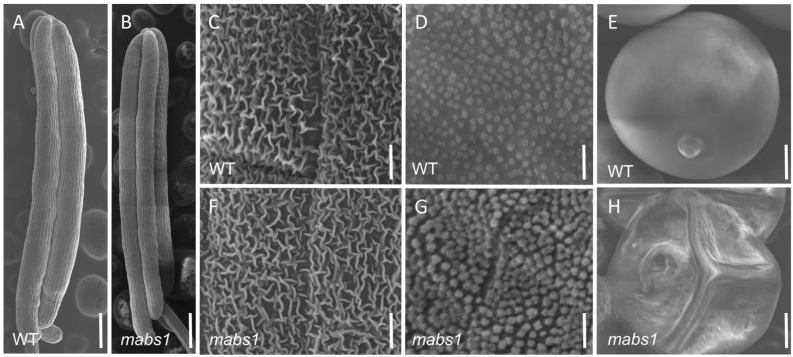
SEM observation of the wild-type and *mabs1*: (**A**,**B**) anthers of wild-type and *mabs1*; (**C**,**F**) anther epidermis of wild-type and *mabs1*; (**D**,**G**) anther inner surface of wild-type and *mabs1*; and (**E**,**H**) pollen grains of wild-type and *mabs1*. Scale bars: 100 μm (**A**,**B**), 10 μm (**C**,**D**,**F**,**G**), and 50 μm (**E**,**H**).

Scanning electron microscopy (SEM) was employed to compare the morphology of the wild-type and *mabs1* anthers. Wild-type anthers were longer and plumper than those of the *mabs1* mutant, which were shriveled (Figure 2A,B). The anther epidermis of the wild-type exhibited a grid-like cuticle structure, which was similarly clear and regular in the *mabs1* mutant (Figure 2C,F). Examination of the anthers’ inner surface revealed densely packed and orderly Ubisch bodies in both wild-type and *mabs1*, with no significant differences observed (Figure 2D,G). Upon dissection, the wild-type anthers contained round and plump pollen grains, while the *mabs1* anthers contained shriveled and unfilled pollen grains (Figure 2E,H). These findings suggest that anther development in *mabs1* is normal but the pollens are sterile.

### 3.3. Semi-Thin Section of mabs1 Anthers

**Figure 3:**
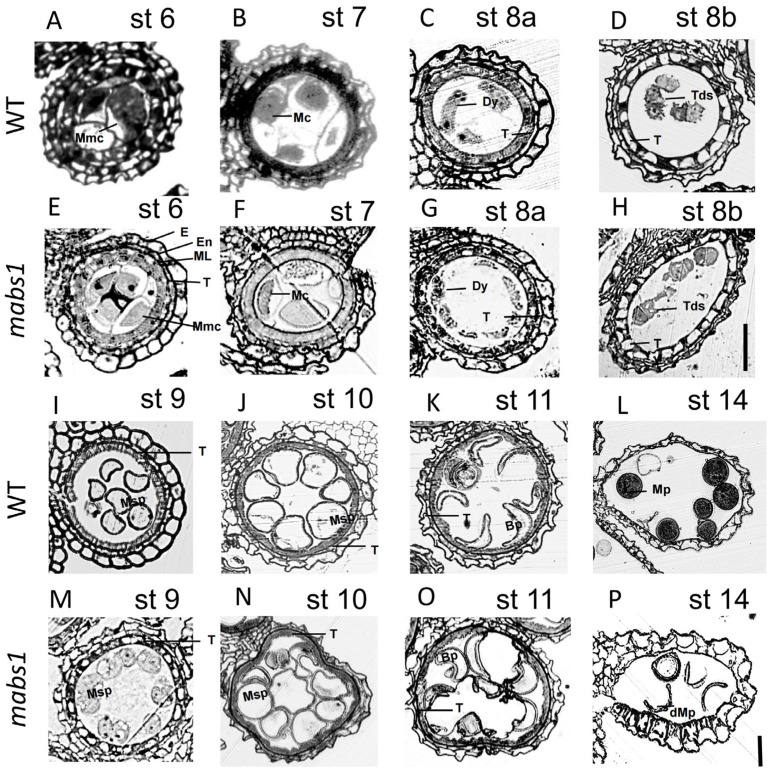
Semi-thin section of wild-type and *mabs1*: (**A**–**D**,**I**–**L**) semi-thin sections of wild-type anthers from st6 to st14 and (**E**–**H**,**M**–**P**) semi-thin sections of *mabs1* anthers from st6 to st14. BP: binuclear pollen; dMp: abnormal mature pollen; Dy: dyad cell; E: epidermis; En: endothelium; ML: middle layer; MP: mature pollen; Msp: microspore mother cell; T: tapetum; and Tds: tetrad. Scale bars: 200 μm (**A**–**P**).

To further investigate the changes in anther tissue structure following the *mabs1* mutation, semi-thin sections were prepared from anthers at various developmental stages. From stages 6 to 8b, the wild-type microspores were undergoing meiosis to form tetrads, with the tapetum beginning to condense and degrade. At this stage, no significant differences were observed between the *mabs1* mutant and the wild-type (Figure 3A–H). At stage 9, the wild-type tapetum continued to degrade and condense, becoming thinner and more irregular, and we observed the same variation in *mabs1* (Figure 3I,M). At stage 10, the wild-type tapetum further degraded, the microspores began to enlarge and vacuolate, and *mabs1* had a consistent trend (Figure 3J,N). At stage 11, the degradation of the tapetum in both the wild-type and *mabs1* increased, with the wild-type microspores taking on a sickle shape (Figure 3K,O). At stage 14, the wild-type microspores were normally filled with starch, forming fertile pollen grains, whereas no completed filling was observed in *mabs1*, as *mabs1* pollen shriveled and deformed, leading to sterility (Figure 3L,P). These results indicate that the tapetum degradation process in *mabs1* is normal, but the pollen fails to fill properly during the final stage, resulting in sterility.

### 3.4. Callose Staining in mabs1

**Figure 4:**
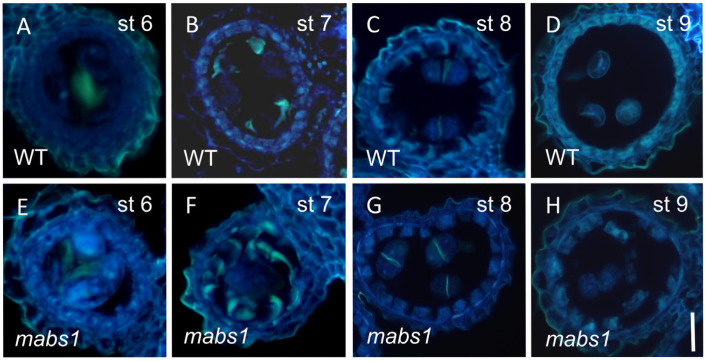
Callose staining of wild-type and *mabs1*: (**A**–**D**) callose staining of wild-type anthers from stages st6 to st9; and (**E**–**H**) callose staining of *mabs1* anthers from st6 to st9. Scale bars: 200 μm (**A**–**H**).

Callose, a substance which encapsulates microspores before mitosis and plays a role in initiating meiosis and protecting microspores, can also lead to microspore abortion when abnormal [33]. To observe whether callose was abnormal in the *mabs1* mutant, 0.1% aniline blue was used for staining. At stage 6, callose was present in the middle of the microspore mother cells, filling the anther locules, with the wild-type showing slightly stronger callose signals than the *mabs1* mutant (Figure 4A,E). At stage 7, the wild-type callose signals were concentrated around the dyads, while the *mabs1* mutant exhibited weaker signals (Figure 4B,F). At stage 8, as the dyads further divided into tetrads, the callose gradually decreased, with a small amount of callose signal present between the two tetrads, showing no significant difference between the wild-type and *mabs1* mutant (Figure 4C,G). At stage 9, the callose was completely degraded, and the tetrads were released to free microspores, with no callose signals observed in either the wild-type or *mabs1* mutant (Figure 4D,H). These results suggest that callose synthesis is reduced in the early stages of the *mabs1* mutant.

### 3.5. Meiotic Chromosome Behavior in mabs1 Mutant

**Figure 5:**
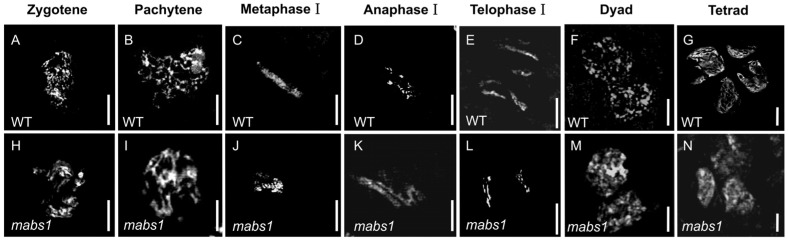
Observation of meiosis behavior of wild-type and *mabs1* chromosome by DAPI staining: (**A**–**G**) wild-type pachytene to tetrad chromosome; and (**H**–**N**) *mabs1* pachytene to tetrad chromosome. Scale bars: 20 μm (**A**–**N**).

Meiosis is a process that both pollen and ovule development must undergo, and abnormalities in meiosis often lead to the sterility of both male and female gametophytes. To observe the meiotic behavior in the *mabs1* mutant, anthers from the meiotic phase were prepared for pollen chromosome analysis and stained with DAPI. It was found that the *mabs1* pollen mother cells showed no significant differences compared to the wild-type during the leptotene to diakinesis stages, entering meiosis normally and forming dyads through homologous recombination (Figure 5A–F,H–M). However, during the formation of tetrads, *mabs1* failed to produce normal tetrads, with only a few abnormal tetrads showing uneven chromatin division (Figure 5G,N). These phenotypic observations indicate that meiotic abnormalities in *mabs1* lead to the loss of fertility in both pollen and female gametophytes.

### 3.6. Fine Mapping of mabs1

**Figure 6:**
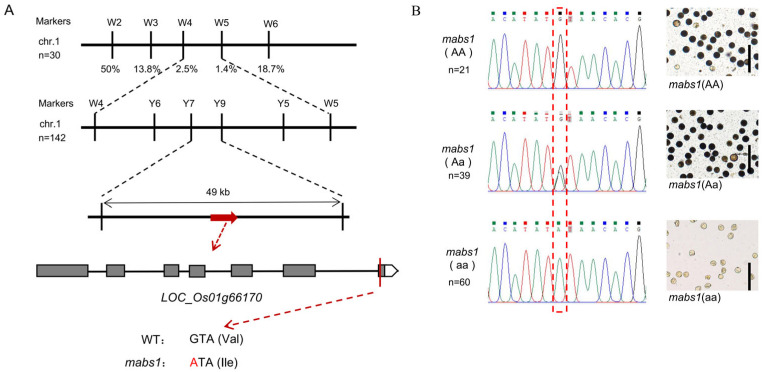
Fine mapping of *MABS1*: (**A**) preliminary positioning and fine positioning of *mabs1*; and (**B**) wild-type and *mabs1* mutation site sequencing results and pollen I_2_-KI staining.

Since *mabs1* is a male–female sterility mutant, the mixed fertile plants were selected from the lines with sterile *mabs1* phenotypic separation as the male parent to cross with Jinhui 10 to obtain the F_1_ generation, as the F_1_ generation was entirely fertile, indicating that the sterility trait was recessive. Self-pollination of the F_1_ generation allowed us to obtain the F_2_ generation, in which 444 fertile wild-type and 142 sterile mutant plants were observed. An χ^2^ test showed that the separation ratio conformed to 3:1, suggesting that the sterile phenotype was controlled by a single recessive nuclear sterility gene (Table 1). DNA from 10 fertile and 30 sterile plants in the F_2_ generation was used for gene pool analysis with SSR primers distributed across the 12 chromosomes. The lowest recombination rates were observed at molecular markers W4 and W5 on chromosome 1, at 2.5% and 1.4%, respectively, indicating a high linkage with the mutation site (Figure 6A). Further development of polymorphic molecular markers between W4 and W5 and analysis with 142 mutant plants from the F_2_ population narrowed the interval to a 49 kb region between Y7 and Y9. To further identify the gene containing the *mabs1* mutation, the Mut-map method based on genome resequencing was used to screen for mutation sites within the interval. A single base substitution was identified in an exon of *LOC_Os01g66170*, changing from "G" to "A" and corresponding to an amino acid change from Val to Ile (Figure 6A). To validate this result, primers specific to this mutation site were designed, and 60 wild-type and 60 mutant plants from the parental generation were sequenced. All wild-type plants had the AA (n = 21) or Aa (n = 39) genotype, while all mutant plants had the aa (n = 60) genotype, which was confirmed by I_2_-KI staining (Figure 6B). Therefore, *LOC_Os01g66170* was designated as the candidate gene for *MABS1*.

### 3.7. Conservative Analysis of MABS1 Sequence

**Figure 7:**
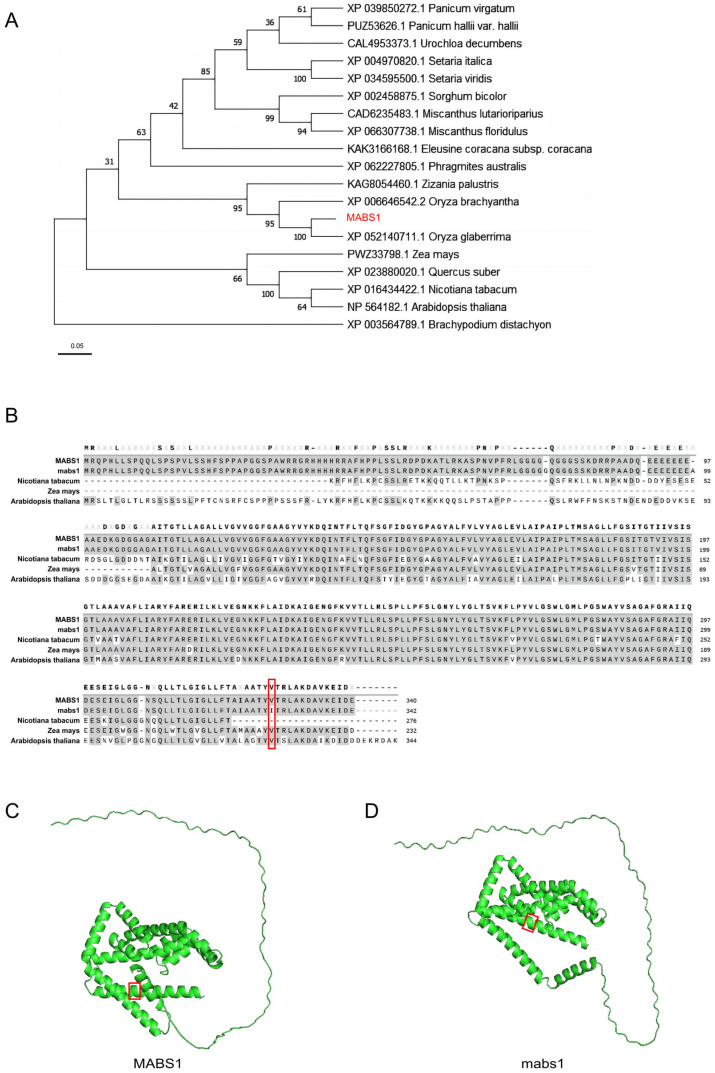
MABS1 sequence analysis: (**A**) phylogenetic tree of MABS1 and its homologous proteins constructed using the MEGA X neighbor-joining method, with bootstrap values indicated by numbers on the branches; (**B**) homologous sequences of MABS1 in *Zea mays*, *Nicotiana tabacum*, and *Arabidopsis thaliana*, downloaded from NCBI and analyzed for homologous segments using SnapGene, with alignment results showing more than 50% homologous amino acids marked at the top of the sequence, and the region enclosed by a red box representing the amino acids at the mabs1 mutation site; and (**C**,**D**) three-dimensional protein structure prediction of MABS1 and mabs1, performed using AlphaFold, with the region enclosed by a red box representing the amino acids at the mabs1 mutation site.

After obtaining the candidate genes, to further analyze their functions, we utilized data from the NCBI to search for MABS1-like proteins. We constructed a phylogenetic evolutionary tree using the neighbor-joining method and found that homologs of MABS1 exist in monocotyledonous plants such as *Oryza glaberrima*, *Zea mays*, and *Zizania palustris*, with a high homology. Additionally, homologs are also present in dicotyledonous model plants *Arabidopsis thaliana* and *Nicotiana tabacum*, indicating that MABS1 is conserved in plant evolution (Figure 7A). We further selected the homologous protein sequences of MABS1, mabs1, *Zea mays*, *Nicotiana tabacum*, and *Arabidopsis thaliana* for sequence alignment using SnapGene. We discovered that the sequence from the 109th amino acid of MABS1 onwards is highly similar to the sequences of the other homologs, suggesting that this region is highly conserved evolutionarily. It was also observed that the mabs1 mutation site was located on a conserved amino acid (Figure 7B). We used AlphaFold to predict the structure of MABS1 and mabs1 proteins and found no significant structural changes between them (Figure 7C,D).

### 3.8. Subcellular Localization of MABS1

**Figure 8:**
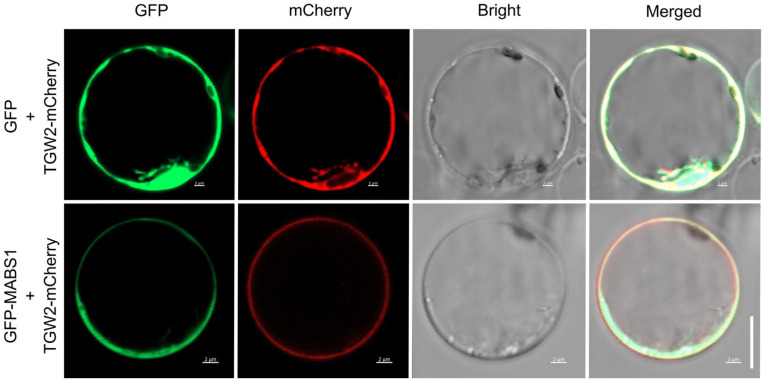
Subcellular localization of MABS1. Subcellular localization of 35S::GFP and GFP-MABS1 in rice protoplasts. TGW2-mCherry was used to indicate the PM. Scale bars: 10 μm.

In order to further confirm that MABS1 belongs to the SNARE protein family, we examined the subcellular localization of the MABS1 protein. Previous studies in Arabidopsis found that most R-SNARE proteins are localized in the plasma membrane (PM) and the trans-Golgi network (TGN). We selected the reported PM-localized protein TGW2 as a marker. By co-transforming rice protoplasts with GFP-MABS1 and GFP-mabs1 along with TGW2-mCherry for transient expression, we observed that the green fluorescence signal of MABS1 largely overlapped with the red fluorescence signal, indicating that MABS1 was localized to the PM (Figure 8), consistent with the localization of the reported SNARE protein.

### 3.9. Gene Expression Analysis

**Figure 9:**
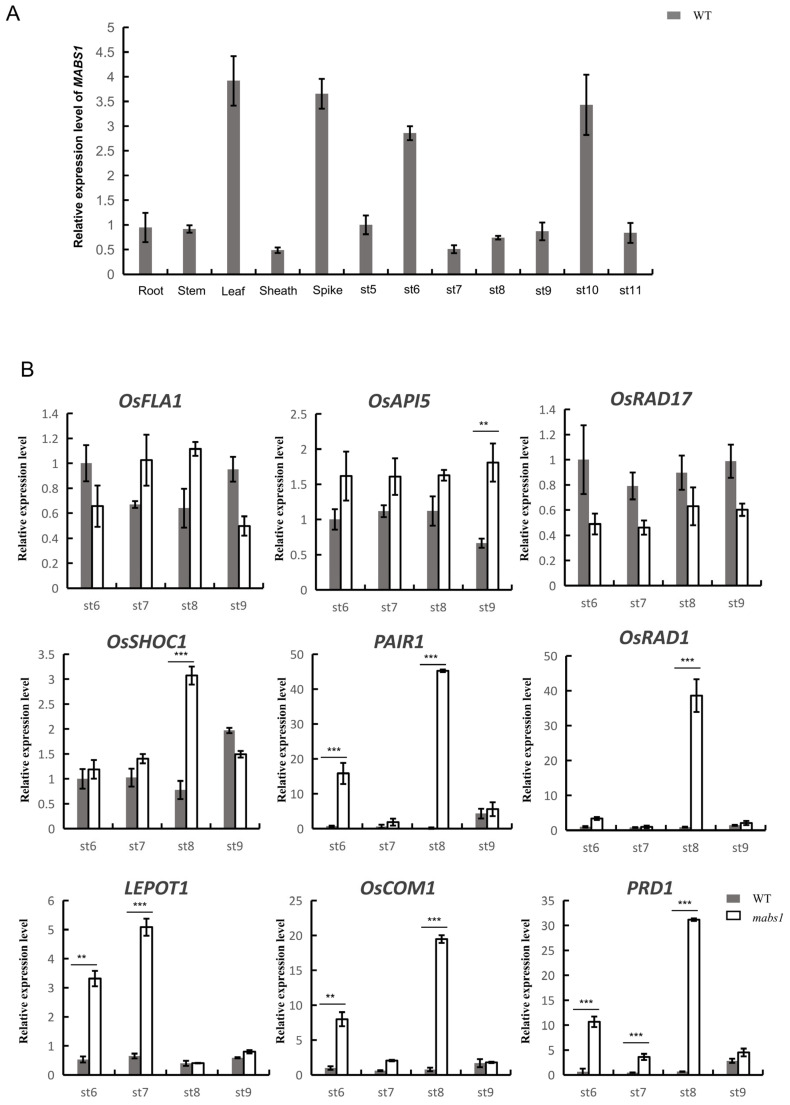
RT-qPCR analysis: (**A**) expression level of *MABS1* in different parts of the heading stage and anther at stages 5-11 in the wild-type; and (**B**) expression levels of related genes in wild-type and mutant *mabs1* at stages 6–9. Data are the means ± SD, n = 3. ** *p* < 0.01 and *** *p* < 0.001.

To analyze the expression levels of *MABS1* during various stages of panicle development in rice, RNA was extracted from the wild-type spikes from stages 5 to 11 and multiple tissue sites during the heading period and reverse transcribed to cDNA. RT-qPCR analysis was performed to assess the expression levels of *MABS1*, which showed that *MABS1* was highly expressed in the leaves and the anthers, and the expression level was higher at stage 6 and stage 10. (Figure 9A). The phenotypic analysis revealed defects in meiotic behavior in the *mabs1* mutant, suggesting that *MABS1* may influence meiotic processes. To explore the association of *MABS1* with meiosis, the expression levels of the reported meiosis-related genes in the *mabs1* mutant were analyzed by RT-qPCR, along with male sterility genes *EAT1*, *TDR*, and *OsUGE1* as the controls (Figure 9B). The expression levels of *EAT1*, *TDR*, and *OsUGE1* showed no significant differences between the wild-type and the *mabs1* mutant. The expression levels of meiosis-related genes *OsSHOC1*, *LEPOT1*, *OsCOM1*, *PRD1*, *PAIR1*, and *OsRAD1* were significantly increased in the *mabs1* mutant. This indicated that *MABS1* is closely related to the meiosis pathway (Figure 9B).

## 4. Discussion

The *mabs1* mutant exhibits no significant differences from the wild-type during the vegetative growth phase but shows complete sterility during the reproductive growth phase. Further observations revealed that the *mabs1* mutant is completely infertile in both pollen and female gametophytes (Figure 1; Table S1). The fertility development of male and female gametes in rice is a rigorous and precise biological process involving a multitude of genes. Male gametes differentiate from sporogenous cells to form microspore mother cells, which then undergo meiosis, tapetum degradation, pollen wall development, and pollen filling to produce mature pollen. Female gamete development, on the other hand, starts from the differentiation of sporogenous cells into megasporocyte mother cells, followed by meiosis, ovule development, and mitosis to form mature female gametophytes [34,35]. Given that both male and female gametes in *mabs1* are infertile and considering that pollen is easier to study and observe, we focused our phenotypic observations primarily on pollen. Previous research indicated that abnormalities in anther wall formation, tapetum degradation, and callose formation can lead to pollen abortion. We examined the inner and outer walls of the anthers, the morphology of the tapetum layer at various stages, and the callose content in *mabs1* pollen and found no significant differences from the wild-type. Additionally, staining of the pollen did not reveal any abnormalities in the inner and outer walls. Meiosis, a step experienced by both microspores and megaspores during development, was observed in *mabs1* pollen, which appeared normal until the dyad stage but failed to form tetrad microspores (Figure 5).

Therefore, we conclude that *mabs1* is a mutant with abnormal meiosis, leading to sterility. Subsequent fine mapping identified the candidate gene as an SNARE (soluble N-ethylmaleimide-sensitive factor attachment protein receptor) vesicle protein-encoding gene, *LOC_Os01g66170*, which has not been previously reported for its function, suggesting that *MABS1* is a novel gene regulating meiosis (Figure 6). SNARE proteins are a class of polypeptides with a relatively small molecular weight (200 to 400 amino acids), and their structure is highly conserved among yeast, animals, and plants [36]. Based on the differences in the conserved core amino acids within the SNARE motif, they can also be divided into Q (Gln)-SNARE (usually corresponding to t-SNARE) and R (Arg)-SNARE (usually corresponding to v-SNARE). MABS1 belongs to the R-SNARE proteins [37,38]. Previous studies have reported that plant R-SNARE proteins play an important role in plant growth and development, with relevant reports in pathways such as cell division, reproductive cell development, and biotic and abiotic stress [39,40,41,42]. We analyzed the expression of the candidate gene in various parts of rice and found high expression levels in young panicles, which further supports the possibility that this gene is the target gene. We also examined the expression levels of some reported meiosis-related genes in the *mabs1* mutant and found that the expression levels of multiple meiosis genes significantly increased, while the expression levels of non-meiosis genes remained unchanged compared to the wild-type, confirming the reliability of the observed upregulation (Figure 7). OsSHOC1 is crucial for chromosome exchange (CO) during meiosis in rice [43]; LEPTO1 is essential for the initiation of meiosis, with the *lepto1* mutant’s pollen mother cells’ chromosomes stalling at the pre-leptotene stage, failing to progress to the typical zygotene chromosome configuration [44]; OsCOM1 plays a significant role in promoting homologous chromosome synapsis and meiotic recombination [23]; PRD1 is involved in the formation of DSBs and spindles during meiosis [45]; PAIR1 controls homologous chromosome pairing during meiosis in rice cells, and the absence or mutation of this gene prevents normal pairing during meiosis [21]; finally, OsRAD1 inhibits non-homologous end joining, promoting the accurate repair of meiotic DNA double-strand breaks [46]. 

The candidate gene *MABS1* encodes an R-SNARE vesicle protein involved in vesicle transport. Homologous proteins to MABS1 have not been reported to date (Figure 7 and Figure 8), but there have been considerable research efforts focused on the functions of R-SNARE proteins within the same family. There have been reports of vesicle transport proteins affecting the fertility of both male and female gametes; for example, the Arabidopsis R-SNAER protein YKT61, when knocked out, significantly reduces the fertility of both pollen and ovules, with mononuclear pollen failing to undergo mitosis to form two-nucleate pollen [41]. Meiosis also relies on vesicle transport to deliver numerous protein signals to guide its normal progression, and the loss of *mabs1* function may disrupt the normal transport of proteins required for these processes, leading to the transcriptional upregulation of related genes. The discovery of this gene will enrich the regulatory network of meiosis in rice. The mabs1 mutation site is located in the functionally conserved sequence of the protein. Our analysis did not reveal any significant changes in the three-dimensional structure of the protein due to the mutation. Therefore, the specific mechanism by which the mutation at this site leads to the loss of function of mabs1 and subsequently causes sterility still requires further exploration.

## Figures and Tables

**Table 1:** Genetic analysis of mutant *mabs1*.

Population	Total Plant Number	Fertile Plants	Sterile Plants	Segregation Ratio	*p*-Value	χ^2^ 3:1
(JH10 × MABS1(Aa)) F2	586	444	142	3∶1	*p* =0.975 > 0.05	0.0012

## Data Availability

Information on candidate genes is available at the China Rice Data Center (https://www.ricedata.cn/, accessed on 8 August 2024) and National Center for Biotechnology Information (https://www.ncbi.nlm.nih.gov/, accessed on 8 August 2024), under accession codes *LOC_Os01g66170* and *LOC4324879*.

## Supplementary Materials

The following supporting information can be downloaded at https://www.mdpi.com/article/10.3390/cimb46110773/s1: Figure S1: Statistics of plant height, blade width, effective tiller number, and seed setting rate of wild-type and *mabs1* mutant; Figure S2: Wild-type and *mabs1* pollen auramine staining and calcium fluorescent white staining; Table S1: Statistics of seed setting rate of wild-type pollen with *mabs1* female gametes’ hybridization; and Table S2: Primers used in this study.
