# Supplementary material for: Identification and Gene Fine Mapping of the Bisexual Sterility Mutant Meiosis Abnormal Bisexual Sterility 1 in Rice
Source: Curr Issues Mol Biol. 2024 Nov 14;46(11):12978–93. doi: 10.3390/cimb46110773 (PMC11592856; doi:10.3390/cimb46110773)
Supplement: Supplementary file 1 [file cimb-46-00773-s001.zip › cimb-3269898-supplementary.pdf]

Identification and Gene Fine Mapping of the Bisexual Sterility Mutant *mabs1* in Rice

Supplementary Materials

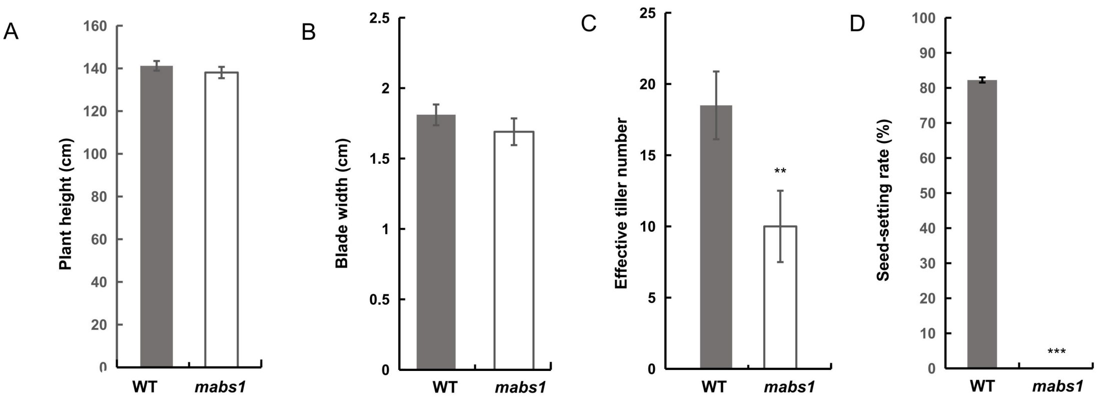

Supplementary Figure S1. (**A**): Plant height of wild type and *mabs1*; (**B**): Blade width of wild type and *mabs1*; (**C**): Effective tiller number of wild type and *mabs1*; (**D**): Statistics of seed setting rate of wild type and *mabs1*. Values are means ± SD of three biological replicates (**p < 0.01, ***p < 0.001; Student's *t*-test).

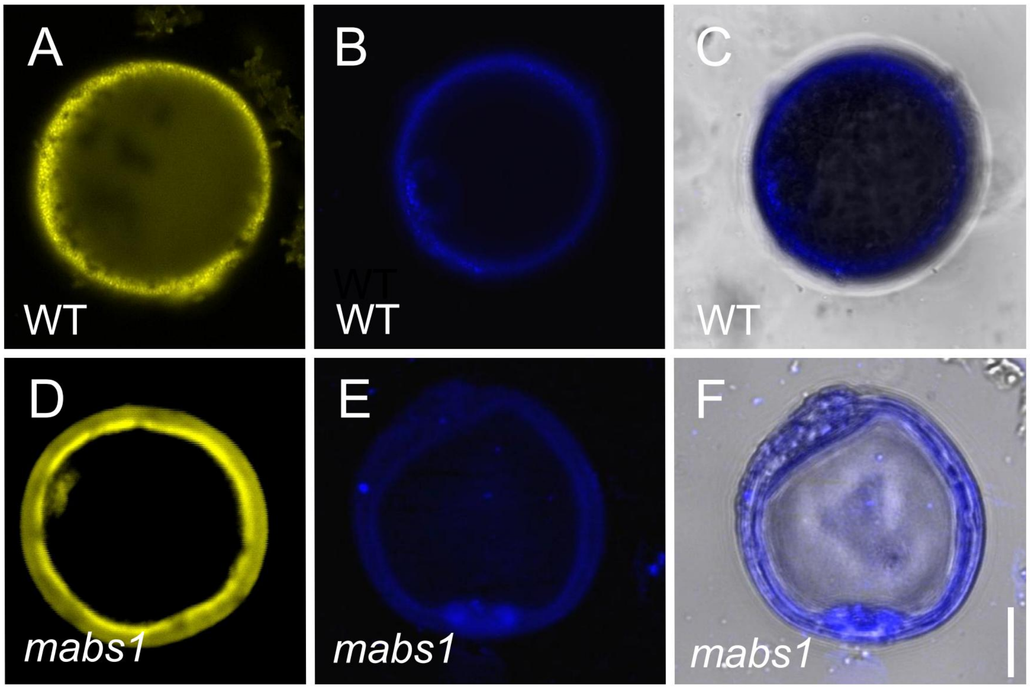

Supplementary Figure S2. (**A**, **D**): The wild-type and *mabs1* pollen Auramine O staining;(**B**, **C**, **E**, **F**): The wild-type and *mabs1* pollen Calcium Fluorescent White staining. Bar: (**A-F**) = 50 μm.

Supplemental Table S1. Statistics of seed setting rate of the wild-type pollen with *mabs1* female gametes hybridization.

♂/♀	Hybrid spikelets	Seed-setting	Seed-setting rate
**WT / WT**	**67**	**23**	**34.3%**
**WT / WT**	**56**	**21**	**37.5%**
**WT / WT**	**69**	**25**	**36.2%**
**WT / *mabs1***	**44**	**0**	**0%**
**WT / *mabs1***	**47**	**0**	**0%**
**WT / *mabs1***	**40**	**0**	**0%**

Supplemental Table S2. Primers used in this study

Use	Primer name	Forward sequence (5′–3′)	Genomic Location*	Reverse sequence (5′–3′)	Genomic Location
Fine Mapping	W2	GCAACTTTAGTACCGGTTCTCTC	1:31106046-31106068	AGCAAAAGAATGTTGAAGCCC	1:31106196-31106216
	W3	ATTGTCGACCACACTACGCA	1:35685365-35685384	AAACTCAGGAAACAGCCAAG	1:35685485-35685504
	W4	TAGCCACCAGGAATTAATCAT	1:36508724-36508744	ACTTATTCCGTCCATAGTAA	1:36508874-36508893
	W5	TGATTATCGTGGACTAAAAT	1:38547881-38547900	GGCTCAATGCCATAGGTCAG	1:38548131-38548150
	W6	GCCAAAGAGGGCATAAATAC	1:40803487-40803506	AGTTCAAGGGTGTAATATAC	1:40803707-40803726
	Y5	TCCTGCCGCCGAACGCCATG	1:38520104-38520123	AGCCCAAAGTGCACGAATCC	1:38520324-38520343
	Y6	TCCTCGTCGACGGCGAATGG	1:38383370-38383389	AAAGCTTGAATTACTTGTTC	1:38383500-38383519
	Y7	TGATTGCTAGAAAACAAACC	1:38416466-38416485	TTAGATAGAACTAAAGCACT	1:38416596-38416615
	Y9	CTAAGTATGAAACATATAAG	1:38451692-38451711	ACTTACTAGTGCTAGATGGG	1:38451812-38451831
Mutant check	mabs1	GACTTGCAAAGGTATTGACT	1:38437482-38437501	GAGAGGCTCGCCGTCGCGTT	1:38436871-38436890
RT-qPCR	Acting	GACCCAGATCATGTTTGAGACC	3:29074451-29074473	CAGTGTGGCTGACACCATCAC	3:29074810-29074830
	MABS1	ATACGTGTACAAGGACCAG	1:38439903-38439921	GCTGATTGAGACAATAACT	1:38438901-38438919
	OsFLA1	ATGTCCTCCCTCGTCTCCAA	4:28911587-28911606	GTCGGAGACCTCAAGAACGG	4:28911304-28911323
	OsAPI5	CCCCTTTATTTATTGGTGAC	2:12374441-12374460	CCTCTACCACCACGGGATAA	2:12374710-12374729
	OsRAD17	TGGGTATATATCCAAGCTG	3:7509447-7509465	ACAGTCGGTACTTGAGTA	3:7509636-7509653
	OsSHOC1	ATTGATCTGCCTGTGGATC	2:25808267-25808285	CACATTGGCAGATACAGAAGA	2:25808496-25808518
	PAIR1	TGAGGATGTTGAGCGCAAA	3:373118-373136	TGCTTTGAGATCGTCTTGT	3:372711-372729
	OsRAD1	TGTTCATGGAATATGAGTA	6:1760407-1760425	CATACATACATGAATCT	6:1759782-1759798
	LEPOT1	TAACCGCAACAAGAGGCAGT	2:4576427-4576446	TTCTCGATGCCAAGCTGGTT	2:4576268-4576286
	OsCOM1	CGAGCAGAATAAACGGAG	6:24517463-24517480	GCAATCTCGCTCTCTAG	6:24517635-24517651
	PRD1	ATTGTTTGGCTTTGTTG	4:16549019-16549035	CTAAGATCGCTTGCAG	4:16549231-16549246
Subcellular localization	GFP-MABS1	GTACAGATCTTAAAGCGGCATG<br>AGGCAGCCGCACCTCCT		AATGTTTGAACTGCAGCCGGCT<br>ATTCATCGATCTCCTTTA	

*** Genomic Location information https://ensembl.gramene.org/Oryza_sativa/Tools/Blast**
